# Feasibility, accuracy and prognostic value of fully automated speckle tracking analysis-derived left ventricular ejection fraction and global longitudinal strain

**DOI:** 10.1038/s41598-025-26497-w

**Published:** 2025-11-27

**Authors:** Yasufumi Nagata, Tetsuji Kitano, Yosuke Nabeshima, Honami BellTatta, Hajime Miki, Hidehiro Namisaki, Masaharu Kataoka, Masaaki Takeuchi

**Affiliations:** 1 Division of Cardiology and Nephrology, School of Medicine, University of Occupational and Environmental Health, 1-1, Iseigaoka, Yahatanishi-ku, Kitakyushu, 807-8555 Japan; 2 Department of Laboratory and Transfusion, School of Medicine, Hospital of University of Occupational and Environmental Health, 1-1 Iseigaoka, Yahatanishi-ku, Kitakyushu, Japan

**Keywords:** Automated analysis, Echocardiography, Cardiac MRI, Speckle-tracking, Prognosis, Cardiology, Outcomes research

## Abstract

Left ventricular ejection fraction (LVEF) has been widely used for LV systolic assessment. However, it has drawbacks including large observer variability and unsatisfactory detectability of subclinical dysfunction. LV global longitudinal strain (LVGLS) by speckle-tracking on two-dimensional echocardiography (2DE) has been reported to be superior to LVEF. However, it may be influenced by the different methodology (manual versus semi-automated analyses) and biased study cohorts. A fully automated analysis can alleviate drawbacks of the LVEF and allow fair comparison between LVEF and LVGLS. We sought to evaluate the feasibility, accuracy of LVEF, and LVGLS measurements by the novel fully automated 2DE software against manual analysis and cardiac MRI feature-tracking (CMR-FT). Additionally, we tested prognostic utility of LVEF and LVGLS. In consecutive 436 patients undergoing CMR and 2DE, the fully automated analysis had excellent feasibility (97%). The correlation in LVEF and LVGLS between the fully automated analysis and the other two techniques was high (*r* = 0.82–0.95). During a median 26-month follow-up, 65 patients experienced cardiac events. In 422 patients successfully analyzed by the fully automated software (63 patients with cardiac events), both LVEF and LVGLS by the fully automated analysis were associated with cardiac events and their prognostic utility was not inferior to manual analyses. Nested Cox proportional hazard models and net reclassification analyses revealed fully automated analysis-derived LVEF and LVGLS did not have an incremental prognostic value over each other. A novel, fully automated analytical software demonstrated excellent applicability to clinical practice by providing reliable LVEF and LVGLS with comparable prognostic utility.

**Supplementary Information:**

The online version contains supplementary material available at 10.1038/s41598-025-26497-w.

## Introduction

Left ventricular ejection fraction (LVEF) is the most often used index for cardiac function^1,2^. Two-dimensional echocardiography (2DE) is the first-line tool for LVEF assessment because of its excellent availability and cost-effectiveness^3^. Important decisions on diagnosis and therapy have been conducted based on LVEF cutoff values^1,4^ although the LVEF by 2DE has several limitations^5^. In the current era of cardiology, requiring early detection of subtle changes in cardiac function through multiple examinations, major limitations of LVEF include a lack of detectability for early abnormalities and relatively large observer variability^2,6^. The process of manual tracing for LVEF is also becoming an additional shortcoming because multiple echocardiographic monitoring is required in the current settings, particularly in cardio-oncology^6^.

Speckle-tracking echocardiography-derived LV global longitudinal strain (LVGLS) is reliable and useful for evaluating cardiac function and prognostication^7–9^. Among patients with preserved LVEF, LVGLS detects subclinical LV dysfunction and stratifies patients according to risks of adverse outcomes^10–12^. Further, many studies have reported LVGLS showed incremental prognostic value over LVEF in patients with specific diseases conditions^7,11,13^. However, it is questionable as to whether LVEF and LVGLS were compared on the same ground regarding study settings and methodology.

Novel automated speckle-tracking software incorporated with artificial intelligence (AI) technology eliminates measurement variabilities and provides both LVGLS and LVEF at once in approximately 10 s (Supplemental Video). This approach not only yields the benefit of convenience and time savings but also eliminates a critical source of bias inherent in prior studies of LVEF and LVGLS. It allows for the direct comparison between LVGLS and LVEF derived from the same unbiased, automated platform, which has not been able to be conducted in prior studies. Herein, we conducted the study to (1) evaluate the measurement accuracy of LV volumes, LVEF, and LVGLS by using the fully automated 2DE speckle-tracking software against conventional manual analysis and cardiac magnetic resonance feature-tracking (CMR-FT) as references; (2) test prognostic values of LVEF and LVGLS derived by the fully automated analysis and compare them to manual analysis; (3) further compare prognostic values between LVEF and LVGLS and investigate their incremental values over each other.

## Methods

### Study population

We included patients who had clinically indicated CMR and who agreed to undergo echocardiograms on the same day at the University of Occupational and Environmental Health Hospital from January 2014 to December 2020. The exclusion criteria included repeat examinations, age of < 20 years, and poor image quality for analysis. This study protocol was approved by the Institutional Review Board of the University of Occupational and Environmental Health (IRB of UOEH). Due to the retrospective nature of the study, the IRB of UOEH waived the need of obtaining informed consent. This study was also conducted in accordance with the principles of the Declaration of Helsinki.

### Echocardiographic image acquisition and fully automated speckle-tracking analysis

**Fig. 1:**
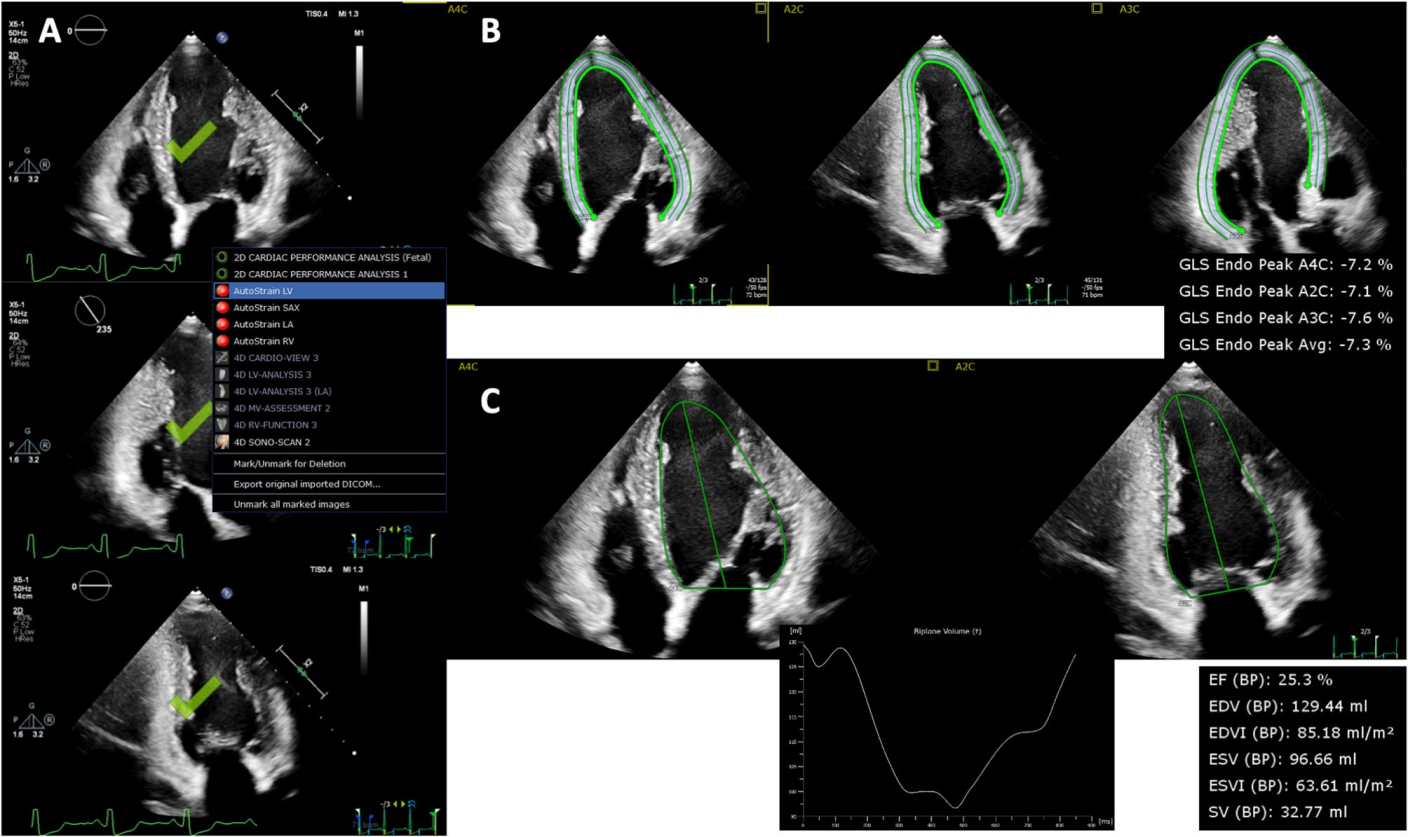
Schema of fully automated speckle-tracking analysis. Three apical images are selected (**A**). The software automatically determines endocardial borders and regions of interest and provide strain values by speckle-tracking analysis (**B**). The software simultaneously provides LV volumes and LVEF on apical two- and four-chamber views (**C**).

All of the subjects underwent comprehensive 2DE by using commercially available ultrasound machines (iE33/EPIQ7G, Philips Healthcare, Andover, Massachusetts; E95, GE Healthcare, Horten, Norway). Echocardiography was performed in the left lateral decubitus position and images were acquired during three cardiac cycles with breath holding. Scan width was adjusted to maximize frame rates. For the fully automated analysis, both LVEF and LVGLS were analyzed by using the commercially available 2DE speckle-tracking software (AutoStrain LV, TomTec Arena 2.50, TomTec, Unterschleissheim, Germany). After the selection of three apical views, the software automatically registered each apical view and determined the LV endocardial border by using a knowledge-based AI algorithm, followed by speckle-tracking analysis during one cardiac cycle for LVGLS. Endocardial longitudinal strain values were used for analyses. LVEF was simultaneously measured from the apical 2- and 4-chamber views (Fig. 1, Supplemental Video). Although the software allows for manual editing, we used the original values of LV parameters derived from the fully automated approach to retain its advantages of rapid analysis and no observer variability. Image quality was evaluated according to the number of visible segments (Excellent: 0–2 segments were poorly visible, fair: 3–5 segments were poorly visible and poor: >5 segments were poorly visible) in the LV 18-segment model. Bad region of interest (ROI) was determined when there was a segment which ROI did not cover the myocardium.

Following the recommendation of guidelines^3^, Conventional measurements of LVEF and LVGLS by manually tracing the endocardial border were performed more than 2 months apart from automated analyses and blinded to its results. LVGLS was analyzed with commercially available software (2D Cardiac Performance Analysis, TomTec Arena 2.50, TomTec, Unterschleissheim, Germany) as previously described^14^.

### CMR image acquisition and feature-tracking analysis

CMR was performed by using a 3.0T scanner (Discovery 750 W or SIGNA premier; GE Healthcare, Milwaukee, WI) with a phased-array cardiovascular coil. Retrospective, electrocardiographically-gated, localizing spin‒echo sequences were used to identify the long axis of the heart. Additionally, steady-state free precession (SSFP) dynamic gradient-echo cine loops were acquired by using parallel imaging techniques during 10- to 15-second breath holding, with the following parameters: slice thickness of imaging planes of 8 mm; field of view of 40 × 40 cm; scan matrix of 200 × 160; flip angle of 50°; repetition time of 2.8 msec; echo time of 1.7 msec; and number of reconstructed cardiac phases of 20 to 30. SSFP CMR images were obtained from stacked LV short-axis views, as well as three apical long-axis views. GLS and LV volumes and EF were obtained by using CMR-FT software (2DCPA MR, TomTec Imaging Systems GmbH, Unterschleissheim, Germany). Using three apical long-axis cine SSFP images, the LV endocardial border at LV end-systole was obtained after clicking three anatomical landmarks. Manual adjustments were performed when needed. Afterwards, the software performed feature-tracking analysis. LV strain and volume curves were obtained on each image and presented as averaged values of LVGLS and LV end-diastolic and end-systolic volumes (LVEDV and LVESV) and LVEF.

### Clinical data and cardiac events

Patient age, sex, anthropometric data, cardiovascular risk factors, and medication information were obtained from the electronic records. Furthermore, the Charlson’s comorbidity index (CCI) was calculated^15^.

Follow-up information was obtained via electronic medical records. If patients were followed by other hospitals, we asked the attending physician to obtain prognostic information. The primary endpoint was a composite cardiac event including cardiac death, sustained ventricular tachyarrhythmia, and heart failure (HF) hospitalization. The follow-up duration was calculated from the date of echocardiography to the event date or censored date.

### Statistical analyses

Continuous variables were expressed as the mean ± standard deviation or median and interquartile range (IQR, 25th percentile-75th percentile) according to the data distribution. Categorical data are expressed as the number and percentage of the total patients. A t-test or Wilcoxon rank-sum test was used for evaluating differences in continuous variables between the two groups. For the categorical variables, the Fisher’s exact test or the chi-square test were used. Additionally, linear correlation and Bland‒Altman analyses were performed to determine the r values, bias, and limit of agreement. Logistic regression analyses were conducted to evaluate the factor affecting poor image quality. Kaplan‒Meier survival analysis was used for analyzing the time to cardiac events between patient groups stratified according to the predefined cutoff values (50% of LVEF and 16% of LVGLS)^16^. The log-rank test was used to evaluate differences between the two groups. Univariable and multivariable Cox proportional hazard analyses were used to evaluate the hazard ratio (HR) for the outcomes. Multicollinearity was assessed via the variance inflation factor. For multivariable Cox proportional hazard analysis, we made nested models including age, NYHA, and Charlson comorbidity index as baseline clinical model. We performed likelihood ratio test between baseline model and baseline model + LVEF(LVGLS), and between baseline model + LVEF (LVGLS) and baseline model + LVEF (LVGLS) + LVGLS (LVEF). Model discrimination was also assessed using Harrell’s concordance index (C-index), and incremental prognostic value was evaluated with the compareC method. Net reclassification improvement (NRI) was calculated to test the incremental discriminative power of LVGLS (LVEF) for risk stratification over the initial model including age, CCI, NYHA functional class, and LVEF (LVGLS). We also evaluated whether the prognostic result by the fully automated analysis was modified by manual analysis. We investigated the prognostic value of LVGLS stratified LVEF subgroup (≥ 50% vs. < 50%). The knotted spline curves of the HR for cardiac events were calculated for LVGLS and LVEF relative to the risk of cardiac events (using LVEF of 50% and LVGLS of 16% as references). Receiver operating curve (ROC) analysis was used to compare prognostic utility of LVEF and LVGLS between two methods (fully automated analysis vs. manual analysis). The intra- and interclass correlation coefficient (ICC) was used for the reproducibility for the fully automated analysis, manual analysis, and CMR-FT in 30 randomly selected patients. Test-retest analyses were performed in different images and different cardiac cycles on the same image acquisition^17^. Analyses were performed by using commercial software (JMP, version 17, SAS, Cary, NC, or R Version 4.2.1, The R foundation for Statistical Computing, Vienna).

## Results

### Study population and feasibility of fully automated strain analyses

Baseline clinical characteristics (*n* = 436) are shown in Table 1. The mean age was 65 years, 282 patients were male (65%), the median CCI value was 5 (IQR: 3 to 6). All patients underwent CMR and echocardiogram within 1 h.

**Table 1:** Baseline characteristics.

Variables	*n* = 436
Demographics	
Age, yrs	65 ± 14
Sex, male	282 (65%)
Body surface area, kg/m^2^	1.63 ± 0.20
Heart rate, bpm	68 ± 14
Systolic blood pressure, mmHg	130 ± 23
Diastolic blood pressure, mmHg	72 ± 13
Clinical history, n (%)	
Etiology	
Dilated cardiomyopathy	48 (11%)
Hypertrophic cardiomyopathy	23 (5%)
Ischemic heart disease	65 (15%)
Myocardial infarction	95 (22%)
Secondary cardiomyopathy	109 (25%)
Cardiac sarcoidosis	37 (9%)
Cardiac amyloidosis	15 (3%)
Hypertensive heart disease	16 (4%)
Chemotherapy related cardiac dysfunction	7 (2%)
Alcoholic cardiomyopathy	6 (1%)
Others	28 (6%)
Valvular heart disease	47 (11%)
Others	49 (11%)
NYHA classification ≧Ⅲ, n (%)	49 (11%)
Charlson comorbidity index	5 (3, 6)
Hypertension	243 (56%)
Diabetes	126 (29%)
Hyperlipidemia	192 (44%)
Coronary artery disease	181 (42%)
Chronic kidney disease	190 (44%)
Medications	
β-blocker	291 (67%)
ACEi/ARB	316 (72%)
Diuretics	205 (47%)
Mineral corticoid receptor blocker	135 (31%)
Warfarin	49 (11%)
Direct oral anticoagulant	55 (13%)

Described as mean$\:\pm\:$SD, median (25%, 75% quartile), or n (%). ACEi, Angiotensin converting enzyme inhibitor; ARB, AngiotensinⅡ receptor blocker; NYHA, New York heart association.

One patient was excluded before LV analysis because of an extremely distorted LV due to severe pulmonary hypertension. A fully automated speckle-tracking analysis was successfully performed on echocardiographic images in 422 patients (422/435, feasibility of 97%), and the analyses were completed in approximately ten seconds, as shown in the Supplemental Video. The software did not set the optimal ROI on the LV endocardial border in 9 patients and/or could not perform adequate speckle tracking in 7 patients, thus resulting in 13 patients being excluded from the analysis. There were significant relationships observed between image quality and the exclusion rate (Supplemental Table S1). Poor image quality was strongly associated with female sex, weight, and body mass index (Supplemental Table S2).

### Comparison of left ventricular parameters between fully automated strain, manual analysis and CMR feature-tracking analyse*s*

**Fig. 2:**
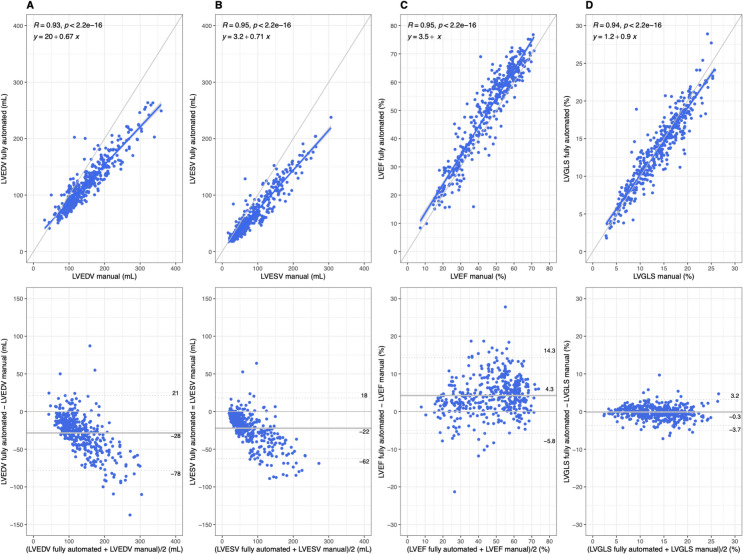
Comparison of left ventricular end-diastolic volume, ejection fraction, and global longitudinal strain between fully automated analysis and manual analysis by linear and Bland-Altman plots. The correlations in LVEDV (**A**), LVESV (**B**), LVEF (**C**), and LVGLS between the fully automated analysis and the manual analysis were excellent (upper figures). Bland-Altman plots showed larger LVEDV (**A**) and LVESV (**B**) were associated with larger differences between measurements while differences in LVEF (**C**) and LVGLS (**D**) were constantly modest to small (lower figures). LVEDV/ESV, left ventricular end-diastolic/end-systolic volume; LVEF, left ventricular ejection fraction; LVGLS, left ventricular global longitudinal strain.

The direct comparison among 2DE fully automated strain, manual analyses and CMR-FT was possible in 395 patients whose images of echocardiogram and CMR were both proper for analyses. LVEDV and LVESV derived by the fully automated analysis were significantly lower than those by manual analysis and CMR-FT (LVEDV: 119 ± 44, 148 ± 61, and 169 ± 76 ml; LVESV: 64 ± 41, 87 ± 55, and 111 ± 68, respectively) (Supplemental Table S3). Although LVEF and LVGLS were statistically different in values among three measurement methods (LVEF: 49 ± 16, 45 ± 15, and 38 ± 13%; LVGLS: 13.6 ± 4.8, 13.8 ± 5.0, and 11.2 ± 4.6%, respectively), these biases were clinically modest between the fully automated analysis and manual analysis (LVEF: 4.3 ± 5.1, LVGLS: -0.3 ± 1.7). Their correlations between the fully automated analysis and manual tracing are excellent (*r* = 0.93–0.95, Fig. 2), and those between the fully automated analysis and CMR-FT were good (*r* = 0.82–0.91, Supplemental Figure S1). Furthermore, the analysis of reproducibility for fully automated measurements expectedly showed zero variability and excellent test-retest analyses (Table 2). Test-retest analyses showed no significant difference between different cardiac cycles in the same acquisition in all LV parameters while there were modest differences between different images (Supplemental Table S4).

**Table 2:** Reproducibility of fully automated, manually edited, manual analyses, and CMR feature tracking.

Intra-rater ICC	LVEDV	LVEF	LVGLS
Fully automated	1	1	1
Manually edited	0.995	0.967	0.993
Manual tracing	0.992	0.977	0.979
CMR feature tracking	0.994	0.989	0.976

Inter-rater ICC	LVEDV	LVEF	LVGLS
Fully automated	1	1	1
Manually edited	0.975	0.950	0.944
Manual tracing	0.977	0.931	0.944
CMR feature tracking	0.960	0.971	0.930

ICC, intraclass correlation coefficients; LVEDV, left ventricular end-diastolic volume; LVEF, left ventricular ejection fraction; LVGLS; left ventricular global longitudinal strain.

### Prognostic value of fully automated strain-derived LVEF and LVGLS

**Fig. 3:**
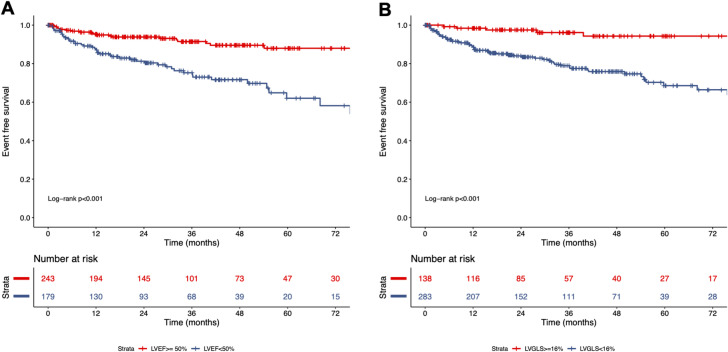
Kaplan-Meier survival curves according to LVEF (**A**) and LVGLS (**B**) derived from the fully automated analysis. LVEF < 50% (**A**) and LVGLS < 16% (**B**) both stratified patients by the event rates.

**Fig. 4:**
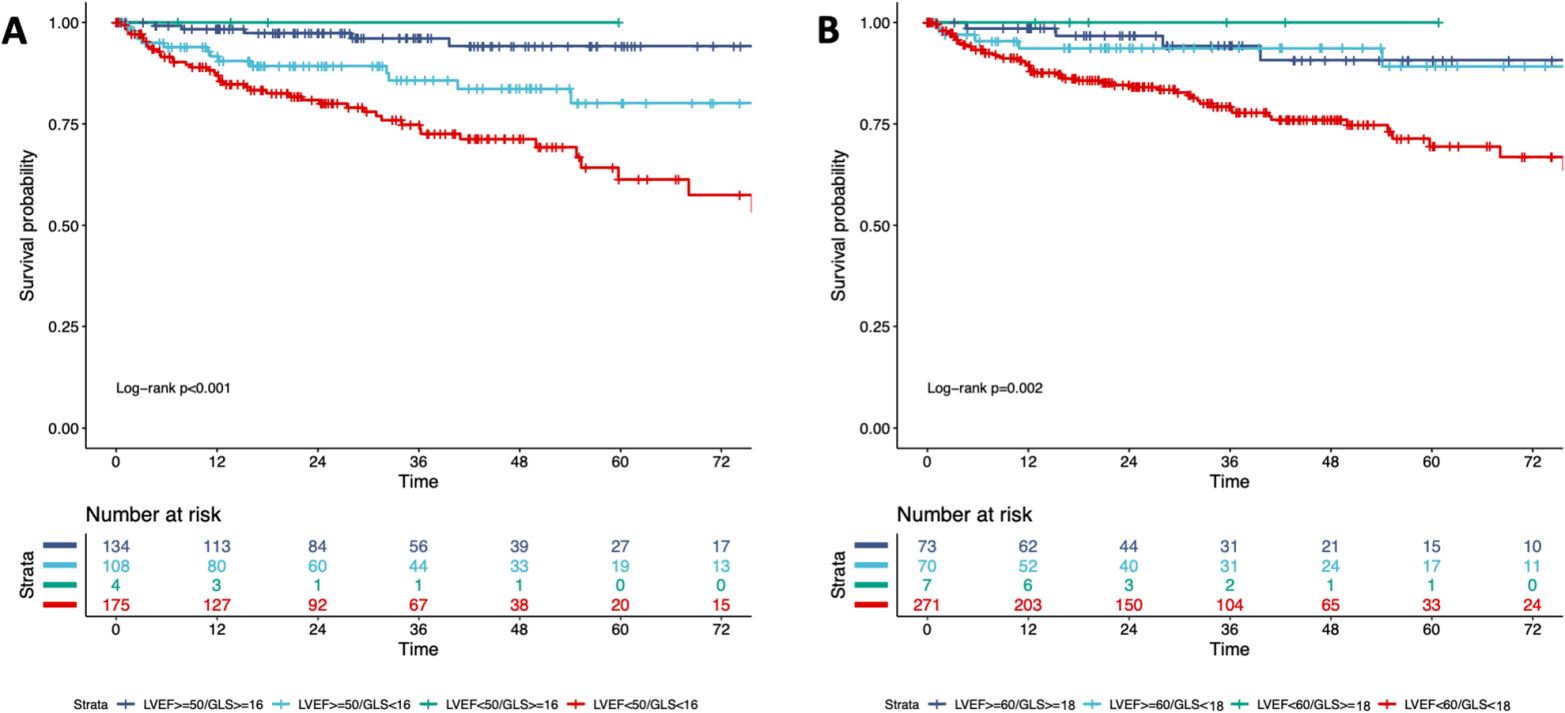
Kaplan-Meier curves according to the combination of cutoff values of LVEF and LVGLS. Kaplan-Meier curves unadjusted by other risk factors showed event free survival events in 4 groups categorized by LVEF and LVGLS cutoffs. Cutoff values are 50% in LVEF and 16% in LVGLS (**A**) and 60% and 18% (**B**).

During a median follow-up time of 26 months (IQR: 12 to 49 months), 65 patients experienced the primary endpoint including 16 cardiac deaths (8 end stage HFs, 3 ventricular fibrillations, 2 sudden cardiac arrests, 2 myocardial infarctions, 1 aortic dissection), 42 hospitalizations due to HF, and 7 ventricular arrhythmias. Patients with events were older, had lower blood pressure, and had higher NYHA classification and CCI values than those without events (Table 3). In 422 patients successfully analyzed by the fully automated software (63 patients with cardiac events), LV volumes were significantly larger, and LVEF and LVGLS were lower in patients with events than those without events. Kaplan‒Meier survival curves revealed both LVEF and LVGLS with predefined cutoff values of 50% and 16% successfully stratified patients according to the risk for adverse outcomes (Fig. 3A, B). The combination of LVEF 50% and LVGLS 16% demonstrated further risk stratification (Fig. 4A). Of 242 patients with LVEF ≥ 50%, patients with LVGLS ≥ 16% had significantly better survival than those with LVGLS < 16% (*p* = 0.022). The univariable Cox proportional hazard analysis demonstrated that both LVEF and LVGLS were significantly associated with cardiac events (Table 3). Due to the concern of collinearity of LVEF and LVGLS, we made separate multivariable Cox models. Both LVGLS and LVEF were significantly associated with cardiac events after adjusting for age, CCI, and NYHA functional class (Table 4).

**Table 3:** Clinical and imaging parameters between groups with or without events and hazard ratios in univariable Cox proportional hazard analysis.

Variables	Event + *n* = 65	Event – *n* = 371	*p*-value	HR (95% CI)	*p*-value
Demographics					
Age, yrs	70 ± 13	65 ± 14	0.002	1.04 (1.02–1.07)	< 0.001
Sex, male	38 (58%)	244 (66%)	0.26	1.31 (0.80–2.14)	0.29
BSA, kg/m^2^	1.58 ± 0.19	1.63 ± 0.21	0.056	0.29 (0.08–0.97)	0.046
Heart rate, bpm	70 ± 15	67 ± 13	0.090	1.02 (0.99–1.03)	0.060
Systolic blood pressure, mmHg	120 ± 19	132 ± 24	< 0.001	0.98 (0.97–0.99)	< 0.001
Diastolic blood pressure, mmHg	66 ± 11	73 ± 13	< 0.001	0.96 (0.94–0.98)	< 0.001
Clinical history, n (%)					
Etiology - ischemic	22 (34%)	135 (36%)	0.69	1.08 (0.64–1.82)	0.76
NYHA classification ≧Ⅲ, n (%)	20 (31%)	29 (8%)	< 0.001	4.72 (2.78–8.01)	< 0.001
Charlson Comorbidity index	6 (4, 8)	4 (3, 6)	< 0.001	1.20 (1.10–1.31)	< 0.001
Hypertension	39 (60%)	204 (55%)	0.45	1.34 (0.81–2.20)	0.25
Diabetes	26 (40%)	100 (27%)	0.032	1.72 (1.04–2.82)	0.034
Hyperlipidemia	32 (49%)	160 (43%)	0.36	1.20 (0.74–1.95)	0.47
Coronary artery disease	28 (43%)	153 (41%)	0.78	1.18 (0.72–1.93)	0.52
Chronic kidney disease	40 (62%)	150 (40%)	0.002	2.71 (1.64–4.49)	< 0.001
Medications					
Calcium channel blocker	12 (18%)	76 (20%)	0.71	0.95 (0.51–1.77)	0.86
β-blocker	48 (74%)	243 (66%)	0.19	1.56 (0.89–2.70)	0.12
ACEi/ARB	57 (88%)	259 (70%)	0.003	3.08 (1.47–6.45)	0.003
Diuretics	44 (68%)	161 (43%)	< 0.001	2.56 (1.52–4.31)	< 0.001
Mineral corticoid receptor blocker	28 (43%)	107 (29%)	0.022	1.84 (1.12–3.00)	0.015
Warfarin	14 (22%)	35 (9%)	0.004	1.96 (1.09–3.56)	0.026
Direct oral anticoagulant	12 (18%)	43 (12%)	0.12	1.70 (0.91–3.19)	0.096
Fully automated (*n* = 422)	*n* = 63	*n* = 359			
Left ventricular end-diastolic volume, ml	127 ± 38	117 ± 44	0.013	1.00 (0.99–1.01)	0.20
Left ventricular end-systolic volume, ml	79 ± 40	61 ± 40	0.001	1.01 (1.00–1.01)	0.004
Left ventricular ejection fraction, %	41 ± 16	51 ± 15	< 0.001	0.96 (0.95–0.98)	< 0.001
LV global longitudinal strain, %	10.8 ± 4.6	14.0 ± 4.7	< 0.001	0.87 (0.83–0.92)	< 0.001
Manual analysis (*n* = 422)					
Left ventricular end-diastolic volume, ml	158 ± 58	144 ± 60	0.096	1.00 (0.99–1.01)	0.19
Left ventricular end-systolic volume, ml	102 ± 56	83 ± 54	0.009	1.00 (1.00–1.01)	0.026
Left ventricular ejection fraction, %	38 ± 15	46 ± 14	< 0.001	0.97 (0.95–0.98)	< 0.001
LV global longitudinal strain, %	11.0 ± 4.9	14.3 ± 4.9	< 0.001	0.88 (0.83–0.93)	< 0.001

Described as mean$\:\pm\:$SD, median (25%, 75% quartile), or n (%). ACEi, Angiotensin converting enzyme inhibitor; ARB, AngiotensinⅡ receptor blocker; BSA, body surface area; HR, hazard ratio; LV, left ventricular; NYHA, New York heart association.

**Table 4:** Multivariable Cox proportional hazard model for cardiac event.

LVEF	Event N	Fully automated analysis			Manual tracing		
variable		HR	95% CI	p-value	HR	95% CI	p-value
Age	63	1.03	1.00, 1.06	0.080	1.03	1.00, 1.06	0.10
Charlson Comorbidity index	63	1.10	0.97, 1.24	0.12	1.10	0.97, 1.24	0.14
NYHA classification	63						
NYHAⅡ		2.16	1.05, 4.43	0.036	2.31	1.13, 4.72	0.022
NYHAⅢ or IV		4.09	1.74, 9.60	0.001	4.42	1.90, 10.3	< 0.001
LVEF	63	0.97	0.96, 0.99	< 0.001	0.97	0.96, 0.99	0.004

LVGLS	Event N	Fully automated analysis			Manual tracing		
variable		HR	95% CI	p-value	HR	95% CI	p-value
Age	63	1.03	1.00, 1.06	0.095	1.03	1.00, 1.06	0.085
Charlson Comorbidity index	63	1.08	0.95, 1.22	0.2	1.08	0.95, 1.22	0.20
NYHA classification	63						
NYHAⅡ		2.24	1.10, 4.57	0.026	2.24	1.10, 4.58	0.027
NYHAⅢ or IV		4.39	1.91, 10.1	< 0.001	4.16	1.78, 9.70	< 0.001
LVGLS	63	0.90	0.85, 0.95	< 0.001	0.91	0.86, 0.96	< 0.001

CI, confidence interval; HR, hazard ratio; LVEF, left ventricular ejection fraction; NYHA, New York heart association.

**Fig. 5:**
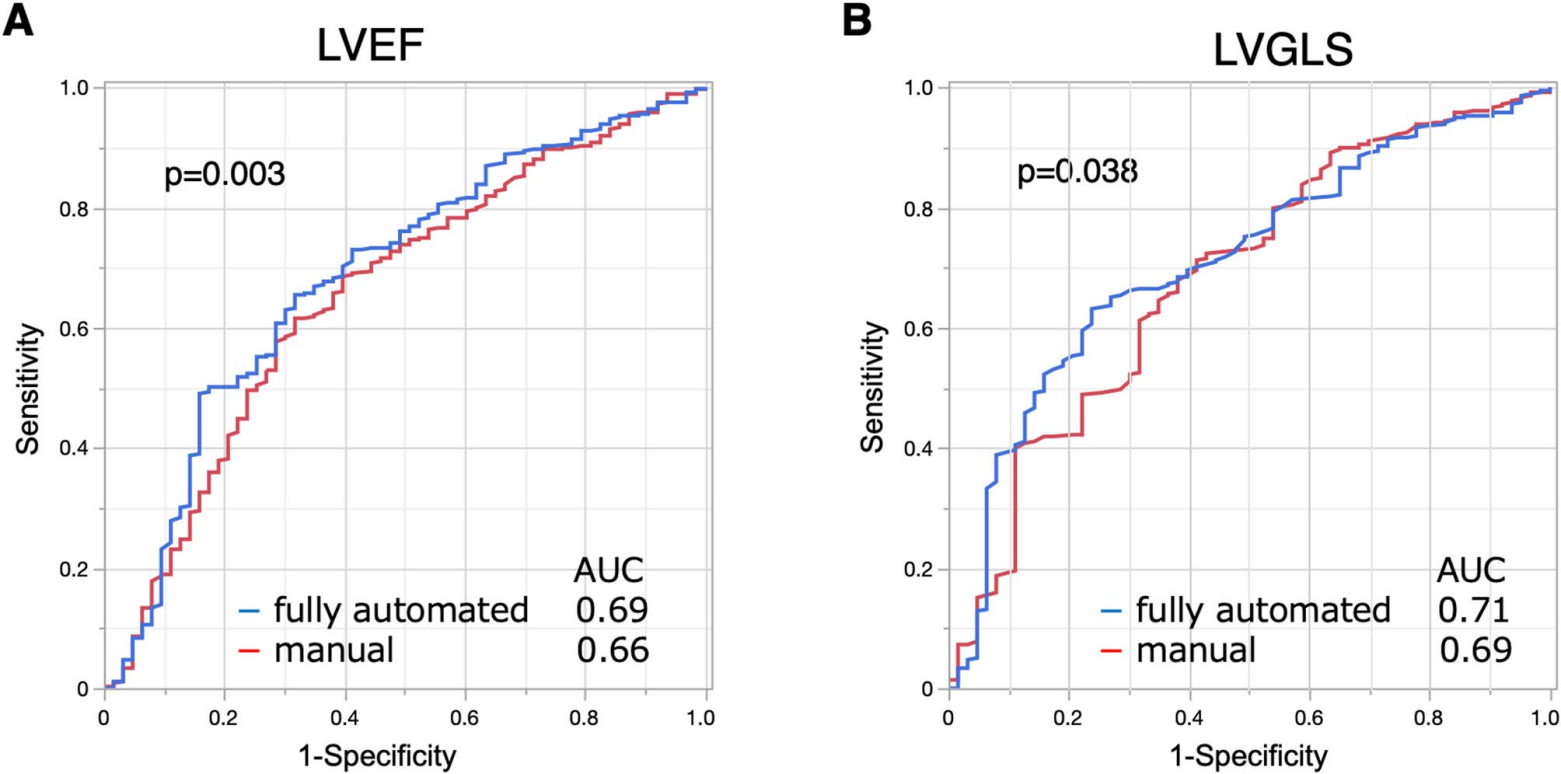
Comparison of area under the curves of receiver operating characteristic in LVEF and LVGLS derived from fully automated and manual analyses. LVEF (**A**) and LVGLS (**B**) derived from the fully automated analysis had significantly higher values of AUC than those by the manual analysis. AUC, area under the curve, LVEF, left ventricular ejection fraction; LVGLS, left ventricular global longitudinal strain.

To validate prognostic utilities of LVEF and LVGLS by the fully automated analysis, we performed the same analysis on the same cohort by standard manual analysis for LVEF and LVGLS (LVEFm and LVGLSm) and evaluated whether risk stratification by the fully automated analysis were modified by manual analysis or not. As shown in Table 4 and Supplemental Figure S2, S3, LVEFm and LVGLSm similarly stratified patients according to MACE with similar hazard ratios. Nested Cox proportional hazard models did not show incremental values of LVEFm and LVGLSm over the models including fully automated analysis-derived LVEF and LVGLS (Supplemental Figure S4). Similarly, reclassification by LVEFm and LVGLSm over the initial model with fully automated analysis-derived LVEF and LVGLS did not improved risk stratification (Supplemental Figure S5). ROC analyses showed significantly higher values of area under the curves both in fully automated analysis-derived LVEF and LVGLS than LVEFm (Fig. 5A) and LVGLSm (Fig. 5B).

We also performed prognostic analysis for CMR feature-tracking derived LVEF and LVGLS. Both LVEF and LVGLS demonstrated prognostic utility similar to that of the fully automated and manually measured LVEF and LVGLS (Supplemental Figure S9, 10).

### Comparison of prognostic power between LVEF and LVGLS

We compared prognostic power between LVEF and LVGLS derived by fully automated analysis under the advantage of no bias and observer variability. While 4 strata using conventional cutoff values of LVEF (50%) and of LVGLS (16%) well stratified patients according to cardiac events (Fig. 4A), using other cutoff values (LVEF 60%, LVGLS 18%) based on those spline curves of HR diminished a benefit of an incremental value of LVGLS in the risk stratification (Fig. 4B, Supplemental Figure S6). In the overall adjusted model including age, NYHA class, Charlson comorbidity index, either LVEF or LVGLS, both LVEF and LVGLS were associated with outcome (Table 4). However, neither reached conventional statistical significance when entered together (LVGLS HR 0.94, 95% CI 0.84–1.06, *p* = 0.31; LVEF HR 0.99, 95% CI 0.95–1.02, *P* = 0.36, variance inflation factor: 4.47 in LVEF and 4.41 in LVGLS).

Likelihood ratio testing confirmed that LVEF or LVGLS improved model performance compared with the baseline clinical model, but further addition of another variable did not provide any significant incremental value (Supplemental Figure S7). Model discrimination was also assessed using Harrell’s concordance index (Table 5). Compared with the baseline clinical model, adding LVEF produced a modest but non-significant increase in C-index (ΔC = 0.027, *p* = 0.16). Adding LVGLS yielded a borderline improvement (ΔC = 0.033, *p* = 0.058). Incorporating both LVEF and LVGLS provided a similar incremental gain (ΔC = 0.032, *p* = 0.093). When models already included LVEF, the addition of LVGLS resulted in a small, non-significant improvement (ΔC = 0.004, *p* = 0.44). Likewise, LVEF did not meaningfully improve discrimination when added to a model already containing LVGLS (ΔC = − 0.002, *p* = 0.70). Overall, although LVGLS tended to show numerically greater improvement than LVEF, none of the differences achieved statistical significance.

**Table 5:** Model discrimination by harrell’s concordance index.

Model	χ^2^ (df)	*p* (χ^2^)	C-index (se)	ΔC	*p* (ΔC)
Baseline (Age, NYHA, CCI)			0.736 (0.036)		
+ LVEF (vs. baseline)	13.59 (1)	< 0.001	0.764 (0.032)	0.027	0.163
+ LVGLS (vs. baseline)	13.78 (1)	< 0.001	0.770 (0.031)	0.033	0.058
+ LVEF + LVGLS (vs. baseline)	14.63 (2)	< 0.001	0.768 (0.032)	0.032	0.093
LVGLS beyond LVEF	1.04 (1)	0.308	0.768 (0.032)	0.004	0.439
LVEF beyond LVGLS	0.85 (1)	0.356	0.768 (0.032)	-0.002	0.701

CCI, Charlson Comorbidity index; LVEF, left ventricular ejection fraction; LVGLS; left ventricular global longitudinal strain; NYHA, New York heart association.

NRI and IDI analysis revealed that addition of LVGLS had not statistically significant NRI (0.16 (95% CI: -0.10–0.43), *p* = 0.23) and IDI (0.0017 (95% CI: -0.0038–0.0073), *p* = 0.54) values compared with age + NYHA + CCI + LVEF model (Supplemental Figure S8). Addition of LVEF also had no statistically significant NRI (0.096 (95% CI: -0.17–0.36), *p* = 0.48) and IDI (0.0026 (95% CI: -0.0047–0.01), *p* = 0.48) values compared with age + NYHA + CCI + LVGLS model (Supplemental Figure S8).

We performed subgroup analysis for LVGLS stratified by LVEF group (LVEF ≥ 50%, LVEF < 50%) (Table 6). In the overall cohort, grouping by LVEF did not show the statistically significant association in multivariable Cox model (HR 1.756, 95% CI 0.81–3.13, *p* = 0.15). LVGLS was borderline prognostic in the adjusted model (HR 0.94, 95% CI 0.86–1.02, *p* = 0.13). To examine whether the prognostic value of LVGLS varied by LVEF group, we performed interaction analyses. LVGLS showed directionally consistent effects across LVEF strata (EF ≥ 50%: HR 0.92, EF < 50%: HR 0.95). There was no evidence of heterogeneity in the prognostic effect of LVGLS between LVEF groups (interaction *p* = 0.78, Table 6). We additionally performed multivariable Cox analysis with four LVEF-LVGLS groups to evaluate prognostic utility to use both functional parameters. After adjustment for age, NYHA, and CCI, both preserved LVEF/reduced LVGLS group (*p* = 0.037) and reduced LVEF/reduced LVGLS group (*p* < 0.001) remained significantly associated with worse outcomes, consistent with the Kaplan-Meier curve (Fig. 4). These results indicate that LVGLS conveys prognostic information consistently across preserved and reduced LVEF, even if it does not demonstrate incremental value beyond LVEF when both are included simultaneously.

**Table 6:** Cox proportional hazards analysis for LVGLS stratified by LVEF group.

Variable	HR (95% CI)	*p*-value
Overall model (fit_base)		
Age (per year)	1.03 (1.00–1.06)	0.100
NYHA (II vs. I)	2.09 (1.02 − 4.29)	0.045
NYHA (III or IV vs. I)	4.18 (1.81–9.66)	< 0.001
CCI (per point)	1.09 (0.96–1.23)	0.17
LVGLS (per 1%)	0.94 (0.86–1.02)	0.13
LVEF < 50% (vs. ≥50%)	1.76 (0.81–3.83)	0.15
Subgroup analyses (fit_int)		
LVGLS (EF ≥ 50%)	0.92 (0.80–1.06)	0.25
LVGLS (EF < 50%)	0.95 (0.85–1.05)	0.31
Interaction (GLS × LVEF group)	—	0.78

CCI, Charlson Comorbidity index; LVEF, left ventricular ejection fraction; LVGLS; left ventricular global longitudinal strain; NYHA, New York heart association.

## Discussion

The novel, fully automated speckle-tracking software simultaneously provides not only LVGLS but also LVEF. To the best of our knowledge, this is the first study to evaluate the measurement accuracy of this software against both manual analysis and CMR, to test the prognostic value of LVEF and LVGLS, and to compare their superiority to each other. The key findings of this study are summarized as follows. (1) The feasibility of the fully automated speckle-tracking analysis was excellent (97%, 422/435). (2) There were strong correlations in LV parameters between the 2DE fully automated analysis and the corresponding values from manual analysis and CMR-FT, although significant differences were observed. (3) LVEF and LVGLS derived by the fully automated analysis had a similar prognostic power for the association with cardiac outcomes with no incremental values over each other. (5) The prognostic power of LVEF and LVGLS by the fully automated analysis were not inferior to manual analysis.

The feasibility and accuracy of automated measurements on echocardiographic images have been important topics and issues^18,19^. In the current study, feasibility for fully automated analysis was high (97%), and the derived measurements had prognostic value comparable to that of manual analysis. However, as anticipated, poor image quality was found to be associated with the software failure. While female sex and increased body mass index can negatively affect image quality, these patients cannot be excluded from echocardiographic examination. Reliability has been improved thanks to steady progress of the technology and introduction of machine-learning-based algorithms^18,20,21^. However, automated software often requires manual editing, especially to match volume measurements^19^. The commercially available latest fully automated analytical software used in this study demonstrated the excellent feasibility and correlation of LV parameters with manual analysis and CMR-FT (even without any manual input). Expectedly, the best observer variability was observed in the fully automated measurements followed by CMR-FT and manual analysis. There were significant difference in value of LV volumes, LVEF, and LVGLS between fully automated analysis and CMR-FT. Difference in LV volumes and LVGLS between echocardiography and CMR have been reported in a previous meta-analysis^22,23^. Several reasons such as different spatial, temporal resolutions, and fundamental image properties between echocardiography and CMR were considered for those differences^2^. For the temporal resolution, frame rates ranged from 50 to 70 frames per second in echocardiography, whereas CMR had approximately 20 or 30 frames per one cardiac cycle. These differences in frame rates could lead to a failure to capture the true end-diastole and -systole, resulting in underestimation of both LVEF and LVGLS by CMR-FT.

The issue of foreshortening in apical views also more frequently occurs in echocardiography than CMR, which caused underestimation of LV volumes, and overestimation of LVGLS(LVEF) by echocardiography. Compared to conventional manual analysis, LV volumes by the fully automated analysis were still small, which suggests it is still difficult even for advanced AI software to accurately determine LV endocardial borders with eliminating trabeculae in echocardiography, which has been reported previously^19^. Considering these conditions possibly causing a considerable underestimation of LV volumes by the fully automated analysis relative to CMR and manual analysis, further technical refinement in the AI-based analysis is required to obtain reliable LV volumes in real-world clinical practice. Therefore, even though fully automated analysis has been continuously improved, examiners and clinicians still need to be trained to possess the ability to judge whether AI-based software adequately performs speckle-tracking analysis and its derived values are clinically plausible. Regarding LV systolic function, the difference in LVEF and LVGLS between the fully automated and manual analyses were clinically modest with excellent correlation (LVEF: 4.3 ± 5.1, *r* = 0.95, LVGLS: -0.3 ± 1.7, *r* = 0.94). When considering the benefit of no bias, no observer variability and time saving in fully automated analysis, these results encourage echocardiographers and clinicians to utilize fully automated analyses in the real-world practice.

The standard concept in the modern cardiology involves early diagnosis followed by early treatment, which requires serial echocardiography examinations^4,6^. LVEF has several limitations, including geometric assumptions, observer variabilities, susceptibility to loading conditions, and less detectability for subtle LV dysfunction^2^. In the past two decades, speckle-tracking echocardiography-derived LVGLS has demonstrated its usefulness and superiority to LVEF in risk stratification and in detecting subclinical LV dysfunction^9,10,13,24^. Park et al. reported that LVGLS has greater prognostic value than LVEF in 4,172 patients with acute HF^25^. Moreover, DeVore et al. showed that two-thirds of HF patients with LVEF ≥ 50% had impaired LVGLS and that LVGLS was well correlated with functional parameters compared to LVEF^26^. Our results also showed that 45% of patients with LVEF ≥ 50% had impaired LVGLS (< 16%). When the LVEF and LVGLS cutoff values were defined as 50% and 16%, both parameters discriminated patients according to event risk and LVGLS further stratified beyond LVEF (Figs. 3 and 4A) which is in conjunction with previous studies^13,24–26^. Further, the fully automated analysis-derived LVEF and LVGLS were not inferior in prognostic utility to those by manual analysis (Fig. 5, Supplemental Figure S4, S5), which supports the potential utility of fully automated analysis in the clinical settings.

Several studies have suggested that LVGLS is robustly better than LVEF^7,8,10,11,13,24,26^. However, those studies have compared LVGLS with LVEF under different methodological conditions and biased cohorts. Specifically, semiautomated speckle-tracking software provided LVGLS, whereas manual LV endocardial border tracing was required for LVEF measurements, which is thought to cause greater observer variability of LVEF than LVGLS. Furthermore, some studies have compared the clinical utility between LVGLS and LVEF in populations where LVEF was already used for the selection of patients^2,25^. Although those studies could identify the incremental utility of LVGLS (but not its superiority to LVEF), which may lead to the misinterpretation that LVGLS is absolutely better. Therefore, we compared the prognostic value between LVEF and LVGLS under the circumstance free from methodological bias thanks to the fully automated analysis. The current software also has advantageous capability to simultaneously measure both LVEF and LVGLS with the same images, which allows for the reliable comparison between LVEF and LVGLS. When using LVEF of 50% as a cutoff in our study cohort, LVGLS could stratify patients according to cardiac events (Fig. 4A). However, when using 60% of LVEF and 18% of LVGLS as cutoff values according to spline curves of HR (Supplemental Figure S6), a LVGLS cutoff value of 18% no longer stratified patients according to risk for cardiac events in patients with LVEF ≥ 60% (Fig. 4B). A nested model of Cox proportional hazard analyses and NRI analysis also did not show incremental values of LVGLS over LVEF (Supplemental Figure S7, 8). This finding may appear inconsistent with previous studies in which GLS was reported to outperform LVEF^2,25,26^. The possible reasons are as follows: (1) we measured both parameters by using fully automated methods, which eliminated the disadvantage of manual LVEF measurement; (2) a fully automated analysis eliminated any measurement variabilities and bias, which may strengthen statistical accuracy; (3) LVEF was not used for subject selection. Among these possibilities, we believe the discrepancy is largely explained by methodological differences. Most prior reports have compared LVGLS measurements obtained using state-of-the-art speckle-tracking software by expert readers with LVEF values obtained through manual tracing or echocardiography report. Most studies did not mention who measured LVEF. This imbalance introduced measurement variability into LVEF assessment and favored LVGLS. In contrast, our study represents a “level playing field”: both LVEF and LVGLS were analyzed automatically by the same platform, without manual intervention. Under these standardized and reproducible conditions, LVEF and LVGLS capture highly overlapping prognostic information, as both reflect global LV systolic function. The absence of incremental value therefore reflects collinearity between the two measures, rather than lack of prognostic utility of LVGLS (or LVEF).

We further examined whether the prognostic effect of LVGLS differed across LVEF subgroups. In stratified analyses, LVGLS showed consistent directional associations with outcome in both preserved and reduced LVEF groups (EF ≥ 50%: HR 0.92 (95% CI 0.80–1.06, *p* = 0.25); EF < 50%: HR 0.95 (95% CI 0.85–1.05, *p* = 0.31), with no evidence of heterogeneity (interaction *p* = 0.78). These results suggest that LVGLS remains prognostic across the spectrum of LVEF, including patients with preserved or borderline LVEF. Although LVGLS and LVEF did not demonstrate incremental value over each other in our cohort, this observation is likely attributable to the statistical limitation of collinearity when including both LVEF and LVGLS as the same form because LVEF and LVGLS are generally well correlated. When patients were categorized using optimal cutoffs of LVEF and LVGLS, LVGLS successfully discriminated patients according to their event risk (Fig. 4A). Consequently, LVGLS is expected to play an important role, especially for patients with preserved LVEF, in real-world clinical settings. Taken together, our findings highlight the importance of considering methodological context when interpreting comparisons of LVEF and LVGLS. When measured under identical automated conditions, LVEF and LVGLS provide similar prognostic information. LVGLS may therefore be viewed not as a redundant parameter, but as a robust, automated, and clinically practical alternative to LVEF for risk stratification. Since number of events was relatively small (*n* = 63), further study including large number of patients should be required to address whether LVGLS is significantly associated with outcomes in LVEF subgroups.

Thus, it is not necessarily a matter of superiority or inferiority between LVEF and LVGLS, but rather a difference in roles. Although LVGLS theoretically better reflects intrinsic systolic function than LVEF^27^, patient clinical courses are influenced by not only systolic function but also other cardiac function and hemodynamic circumstances^1^, which suggests complementary use of both parameters is dispensable to contributes to better clinical practice.

The latest fully automated software used in this study provides both LVEF and LVGLS simultaneously in approximately 10 s, which is another benefit for reduction of measurement time. Thus, introduction of automated measurements can yield the benefit for bias, observer variability and time-demand, which quite meets a current clinical need for early diagnosis and intervention^21,22^. Taken together, findings in this study encourage the adoption of fully automated analysis in real clinical practice to respond to growing demands for echocardiography. Given the findings, including excellent correlation with minimal differences in values from manual analysis, reliable prognostic values, and excellent test-retest analysis in fully automated analysis-derived LVEF and LVGLS, the benefits of fully automated-derived LVEF and LVGLS can be realized in routine clinical settings. These benefits are particularly maximized in the context of functional monitoring for individual patients at risk of progressive diseases and those receiving cardiotoxic drugs. In such scenarios, it may be feasible to replace LVEF and LVGLS measured by conventional methods with those derived from fully automated analysis.

Additional multi-center validation and prospective prognosis studies can confirm its clinical utility and facilitate its widespread adoption. Once integrated into the existing medical infrastructure, fully automated analysis is expected to offer numerous benefits. These include its integration into clinical decision-making workflows, use in the serial monitoring of cardiac function in patients with progressive diseases, and the ability to perform unbiased comparisons across different hospitals. The current rapid progress in AI-based technology promises to expand the application of fully automated analysis to multiple cardiac parameters, including diastolic strain, right heart function, and valve morphology in the future.

### Limitations

We must acknowledge several limitations in this study. First, since this was a single-center, retrospective study using the CMR database, there was an inherent risk of selection bias. However, this did not affect the protocol to evaluate the feasibility and accuracy of the software. An impact of potential selection bias is likely minimal for the purposes of this study to compare the prognostic value between LVEF and LVGLS under the same conditions and methodology. However, to elucidate whether LVGLS or LVEF plays a different role as the prognostic tool in some specific populations, further study including large number of patients is required. Second, there was a significant difference of LV volumes, LVEF and LVGLS by using the fully automated analysis compared to CMR feature-tracking derived values, those differences were diminished when comparing to manual analysis. These findings suggest current AI-based software tends to recognize inner side of the LV myocardium (noncompacted myocardium) as an endocardial border^19^. However, both fully automated analysis-derived LVEF and LVGLS had strong association with cardiac events, which were not modified by manual analysis. Although the imaging dataset of echocardiography and CMR were obtained within 1 h (described in the result section), image itself including a cut-plane was fundamentally different between 2D echocardiography and CMR. Since manual echocardiographic measurements were conducted in the same image with fully automated measurements, the correlation between fully automated analysis and manual analysis could be better than that between fully automated analysis and CMR-FT. Thirdly, we did not assess inter-operator reproducibility. This is a critical consideration in real-world clinical practice, as one of the main sources of variability in echocardiographic measurements is the inconsistency in image acquisition across different operators. While a primary benefit of fully automated analysis is to eliminate observer-based biases in the same image, further studies should be required to address this topic. Fourthly, our results did not demonstrate an incremental value of LVGLS over LVEF as continuous variables in multivariable analysis. This observation may be attributed to the statistical limitation of the collinearity between LVEF and LVGLS and the small event number. However, other analytical information supports the utility of LVGLS in clinical settings. Fifthly, it is unknown as to whether the findings of this study can be extrapolated to other vendors’ products. Although it is quite difficult, sharing with automated analytical algorithms may facilitate automated analyses in broad clinical settings. Lastly, it has not known whether difference in ultrasound machine affects values derived by automated strain analysis although this software is vendor-independent.

## Conclusions

Although there were significant differences in LV volumes, LVEF, and LVGLS among CMR-FT, the conventional manual analysis, and a novel 2DE fully automated analytical software, their correlations were excellent and differences in LVEF and LVGLS between the fully automated analysis and manual analysis were clinically modest. The fully automated analysis-derived LVEF and LVGLS provided comparable prognostic values for the association with adverse outcomes. LVGLS did not show superiority to LVEF in risk stratification but still had the potential to identify patients with impaired longitudinal LV shortening among those with preserved LVEF. The fully automated analytical software may be ready to be introduced into routine clinical workflows with its excellent feasibility, reproducibility, unbiased fashion, potential prognostic value, and rapid analysis time.

## Supplementary Information

Below is the link to the electronic supplementary material.

Supplementary Material 1

Supplementary Material 2

Supplementary Material 3

## Data Availability

All the data generated or analyzed during this study are included in this published article and its supplementary information files.
