# Supplementary material for: Feasibility, accuracy and prognostic value of fully automated speckle tracking analysis-derived left ventricular ejection fraction and global longitudinal strain
Source: Sci Rep. 2025 Nov 27;15:42453. doi: 10.1038/s41598-025-26497-w (PMC12660899; doi:10.1038/s41598-025-26497-w)
Supplement: Supplementary file 3 — Supplementary Material 3 [file 41598_2025_26497_MOESM3_ESM.pdf]

SUPPLEMENTAL MATERIALS

Feasibility, Accuracy, and Prognostic Value of Fully Automated Speckle Tracking Analysis-Derived Left Ventricular Ejection Fraction and Global Longitudinal Strain

Nagata, et al

<u>Supplemental Table</u>

Supplemental Table S1. Relation between image quality and ROI determination/tracking.

		Image quality			
Variables	n	Excellent, n=109	Fair, n=226	Poor, n=100	p-value
Bad ROI	435	2 (1.8%)	0 (0%)	7 (7.0%)	<0.001
Not tracking	435	0 (0%)	1 (0.4%)	6 (6.0%)	0.001

ROI, region of interest

Supplemental Table S2. Association between image quality and patient characteristics

Variables	Odds ratio	95% CI	p value
Age, years	1.10	0.99 - 1.03	0.14
Sex, male, n (%)	0.48	0.30 – 0.75	0.001
Height, cm	1.01	0.99 – 1.04	0.21
Weight, kg	1.03	1.01 – 1.05	<0.001
BMI, kg/m^2	1.14	1.08 - 1.21	<0.001
BSA, kg/m^2	4.20	1.39 – 13.0	0.012
Heart rate, bpm	0.99	0.97 – 1.01	0.22
Systolic blood pressure, mmHg	1.00	0.99 – 1.01	0.40
NYHA≧3, n (%)	1.74	0.90 – 3.28	0.10
Charlson comorbidity index	0.91	0.83 – 0.99	0.035
Hypertension, n (%)	0.82	0.52 – 1.28	0.38
Diabetes, n (%)	1.13	0.69 – 1.83	0.61
Hyperlipidemia, n (%)	0.89	0.57 – 1.40	0.62
Coronary artery disease, n (%)	0.70	0.43 – 1.10	0.12
Chronic kidney disease, n (%)	0.98	0.62 – 1.53	0.92
Chronic obstructive pulmonary disease, n (%)	2.05	0.69 – 8.84	0.22

BMI, body mass index; BSA, body surface area; CI, confidence intervals

Supplemental Table S3. Comparison of LV volumes, LVEF, and LVGLS

	Fully automated	Manual tracing	Bias vs. FA	r value vs. FA	CMR-FT	Bias vs FA	r value vs. FA
LVEDV, ml	119±44	148±61	-29±26*	0.93	169±76	-50±43*	0.88
LVESV, ml	64±41	87±55	-23±21*	0.95	111±69	-46±35*	0.92
LVEF, %	49±16	45±15	4.3±5.1*	0.95	38±13	11±8*	0.87
LVGLS, %	13.6±4.8	13.8±5.0	-0.3±1.7**	0.94	11.2±4.6	2.4±2.9*	0.82

* p<0.001, ** p<0.01

CMR-FT, CMR feature tracking; FA, fully automated analysis

Supplemental Table S4. Test-retest analyses for fully automated analysis with different cardiac cycles and images in echocardiography

Different cardiac cycles						
	Cycle A	Cycle B	Bias	p-value	r	ICC
LVGLS	13.0±3.8	13.1±3.8	0.0±0.6	0.93	0.99	0.987 (0.973, 0.994)
LVEF	46.8±12.5	46.1±12.8	-0.7±2.8	0.17	0.98	0.975 (0.949, 0.988)
LVEDV	124±38	125±39	1.2±4.7	0.18	0.99	0.992 (0.984, 0.996)
LVESV	68±29	69±32	1.9±7.5	0.18	0.97	0.968 (0.935, 0.985)

Different images						
	Image A	Image B	Bias	p-value	r	ICC
LVGLS	13.0±3.8	12.5±3.8	0.5±1.2	0.012	0.96	0.949 (0.897, 0.975)
LVEF	46.8±12.5	45.1±13.2	-1.8±3.6	0.011	0.96	0.952 (0.903, 0.977)
LVEDV	124±38	128±39	4.3±9.4	0.018	0.97	0.965 (0.929, 0.983)
LVESV	68±29	74±33	6.5±9.5	<0.001	0.96	0.933 (0.866, 0.968)

ICC, Intraclass correlation coefficient; LVEDV(ESV), Left ventricular end-diastolic volume (end-systolic volume), other abbreviations previously described.

Supplemental video and figures' legend

Supplemental Figure S1. Comparison of left ventricular end-diastolic volume, ejection fraction, and global longitudinal strain between fully automated analysis and CMR-feature-tracking analysis by linear and Bland-Altham plots.

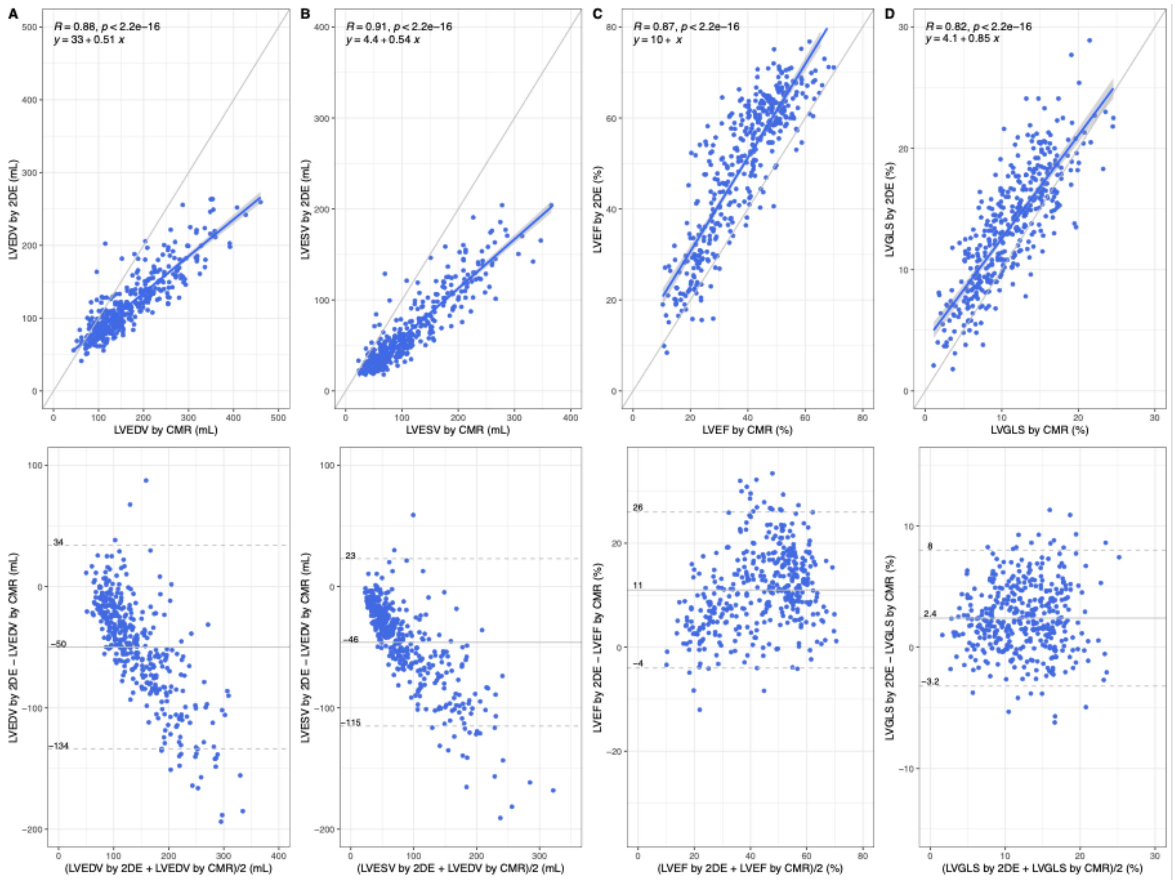

CMR, cardiac magnetic resonance; LV, left ventricular

Supplemental Figure S2. Kaplan-Meier survival curves according to the predefined cutoff values of LVEF (A) and LVGLS (B) derived by conventional manual analysis.

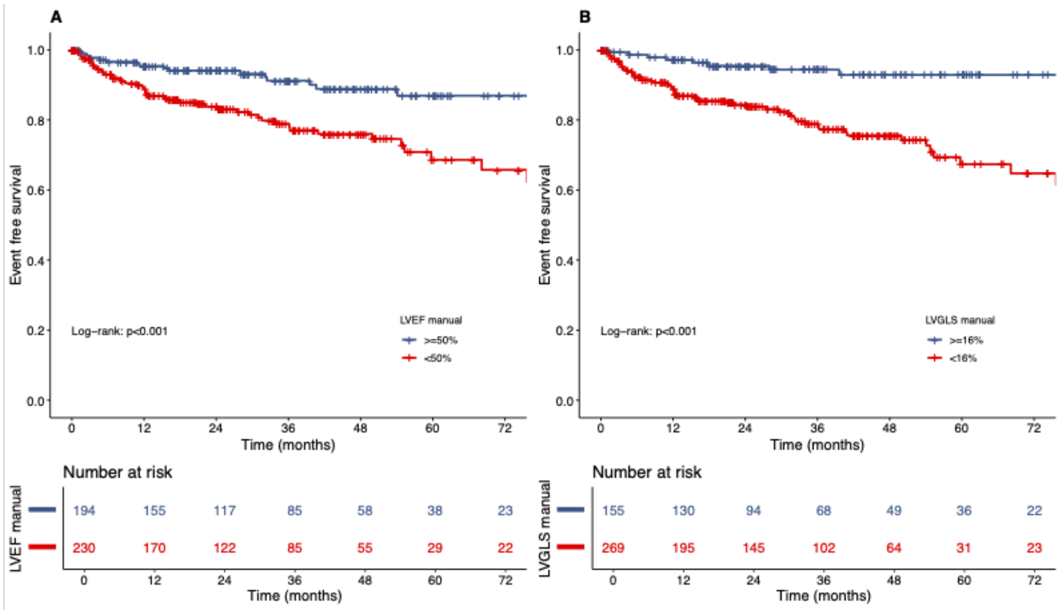

Both LVEF (50%) (A) and LVGLS (16%) (B)stratified patients according to the event rates.

LVEFm, left ventricular ejection fraction derived by manual analysis; LVGLSm, left ventricular global longitudinal strain derived by manual analysis.

Supplemental Figure S3. Kaplan-Meier curves according to the combination of cutoff values of LVEF and LVGLS derived by manual analysis.

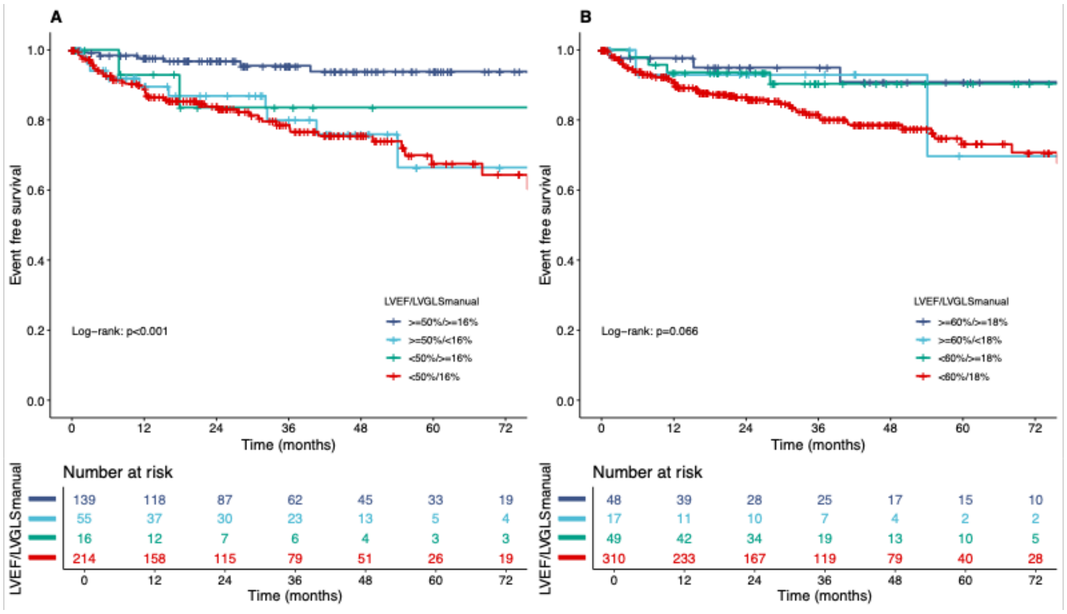

Cutoff values are 50% in LVEF and 16% in LVGLS (A) and 60% and 18% (B)

LVEFm, left ventricular ejection fraction derived by manual analysis; LVGLSm, left ventricular global longitudinal strain derived by manual analysis.

Supplemental Figure S4. Nested Cox proportional hazard models for cardiac events.

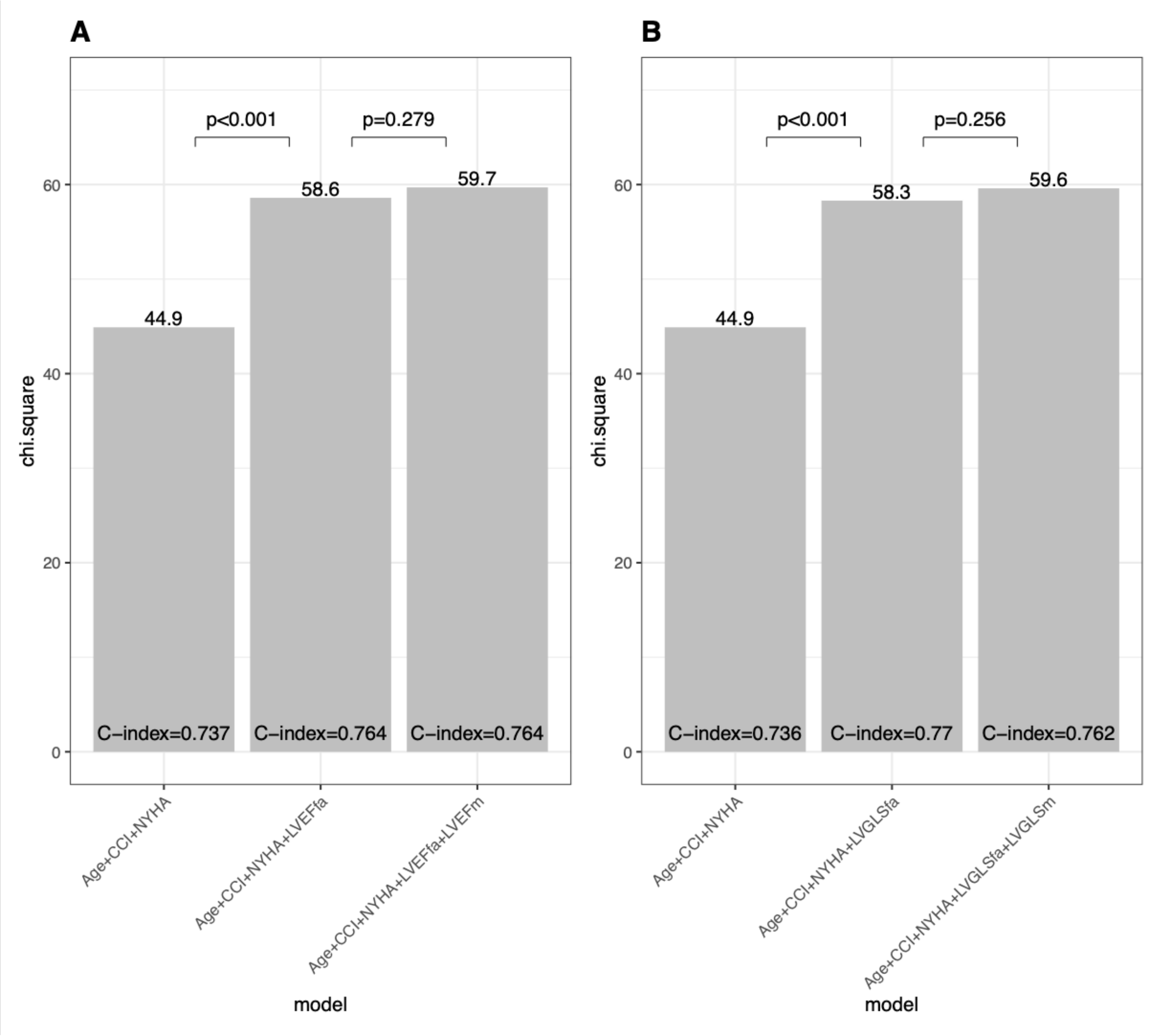

Addition of LVEF derived by manual analysis (LVEFm) on the model that included age, Charlson comorbidity index (CCI), NYHA, LVEF derived by fully automated analysis (LVEFfa) did not increase C-index at the last stepwise. The same trend was observed in LVGLS.

Supplemental Figure S5. Reclassification tables by addition of LVEF and LVGLS derived by manual analysis on the initial model with LVEF and LVGLS by fully automated analysis.

A: LVEF by manual measurement

Reclassification table

Outcome: absent

Initial Model	Updated Model [0,0.047)	[0.047,0.112)	[0.112,0.21)	[0.21,1]	% reclassified
[0,0.047)	95	7	0	0	7
[0.047,0.112)	7	86	8	0	15
[0.112,0.21)	0	6	73	4	12
[0.21,1]	0	0	11	62	15

Outcome: present

Initial Model	Updated Model [0,0.047)	[0.047,0.112)	[0.112,0.21)	[0.21,1]	% reclassified
[0,0.047)	3	1	0	0	25
[0.047,0.112)	0	3	1	0	25
[0.112,0.21)	0	2	16	4	27
[0.21,1]	0	0	1	32	3

Combined Data

Initial Model	Updated Model [0,0.047)	[0.047,0.112)	[0.112,0.21)	[0.21,1]	% reclassified
[0,0.047)	98	8	0	0	8
[0.047,0.112)	7	89	9	0	15
[0.112,0.21)	0	8	89	8	15
[0.21,1]	0	0	12	94	11

NRI(Categorical) [95% CI]: 0.0615 [-0.0377 - 0.1608] ; p-value: 0.22425
NRI(Continuous) [95% CI]: 0.2181 [-0.0472 - 0.4833] ; p-value: 0.10714
IDI [95% CI]: 0.0036 [-0.0023 - 0.0096] ; p-value: 0.22963

B: LVGLS by manual measurement

Reclassification table

Outcome: absent

Initial Model	Updated Model [0,0.0493)	[0.0493,0.109)	[0.109,0.216)	[0.216,1]	% reclassified
[0,0.0493)	95	6	0	0	6
[0.0493,0.109)	4	95	1	0	5
[0.109,0.216)	0	3	75	7	12
[0.216,1]	0	0	6	66	8

Outcome: present

Initial Model	Updated Model [0,0.0493)	[0.0493,0.109)	[0.109,0.216)	[0.216,1]	% reclassified
[0,0.0493)	3	1	0	0	25
[0.0493,0.109)	0	4	1	0	20
[0.109,0.216)	0	0	20	0	0
[0.216,1]	0	0	1	33	3

Combined Data

Initial Model	Updated Model [0,0.0493)	[0.0493,0.109)	[0.109,0.216)	[0.216,1]	% reclassified
[0,0.0493)	98	7	0	0	7
[0.0493,0.109)	4	99	2	0	6
[0.109,0.216)	0	3	95	7	10
[0.216,1]	0	0	7	99	7

NRI(Categorical) [95% CI]: 0.0131 [-0.0477 - 0.0739] ; p-value: 0.67332
NRI(Continuous) [95% CI]: 0.1129 [-0.1542 - 0.3799] ; p-value: 0.40737
IDI [95% CI]: 8e-04 [-0.0025 - 0.0041] ; p-value: 0.63518

(A) Addition of the LV ejection fraction measured by manual analysis (LVEFm) on the initial model with age, Charlson comorbidity index, NYHA functional class, and LVEF by fully automated analysis did not improve risk stratification. (B) Addition of the LV global longitudinal strain by manual analysis (LVGLSm) on the initial model with age, Charlson comorbidity index, NYHA functional class, and LVGLS by fully automated analysis did not improve risk stratification.

Supplemental Figure S6. Knotted spline curves of the association between event hazard ratio and LVEF (A) and LVGLS (B).

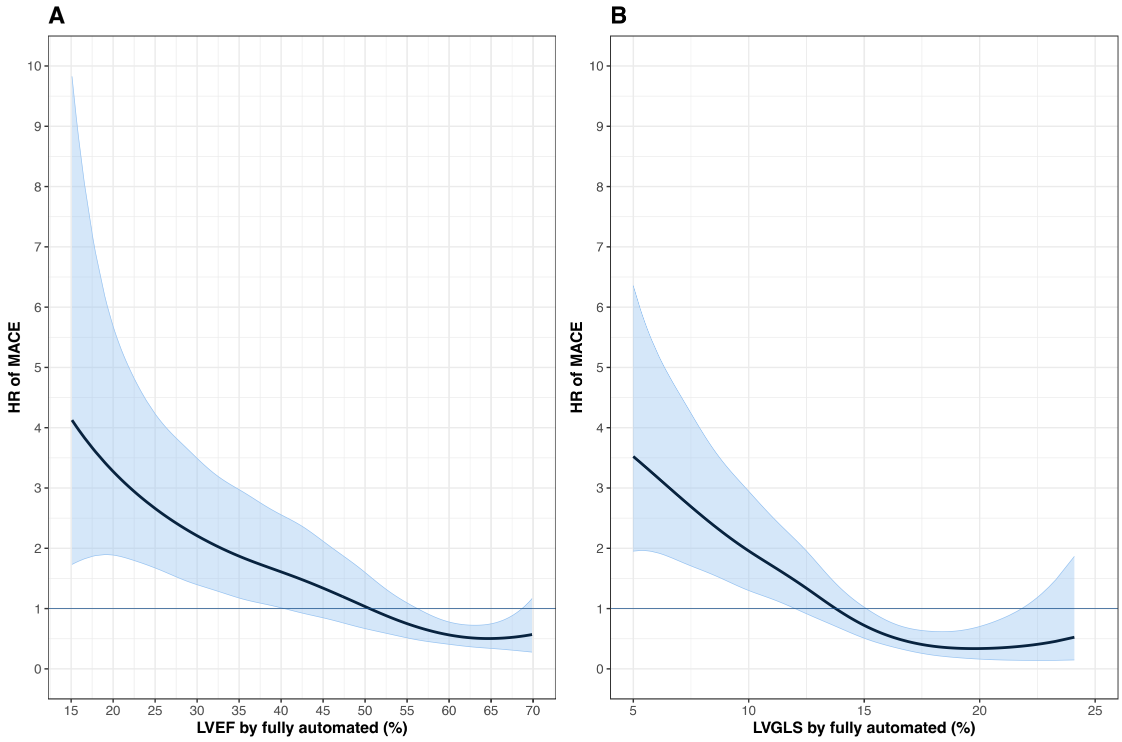

The curve for LVEF revealed a consistent decline of the HR until 60% of LVEF and then almost plateaued (A). The curve for LVGLS also showed a decline of the HR until around 18% and a slight increase thereafter (B).

Supplemental Figure S7. Nested Cox proportional hazard models for cardiac events.

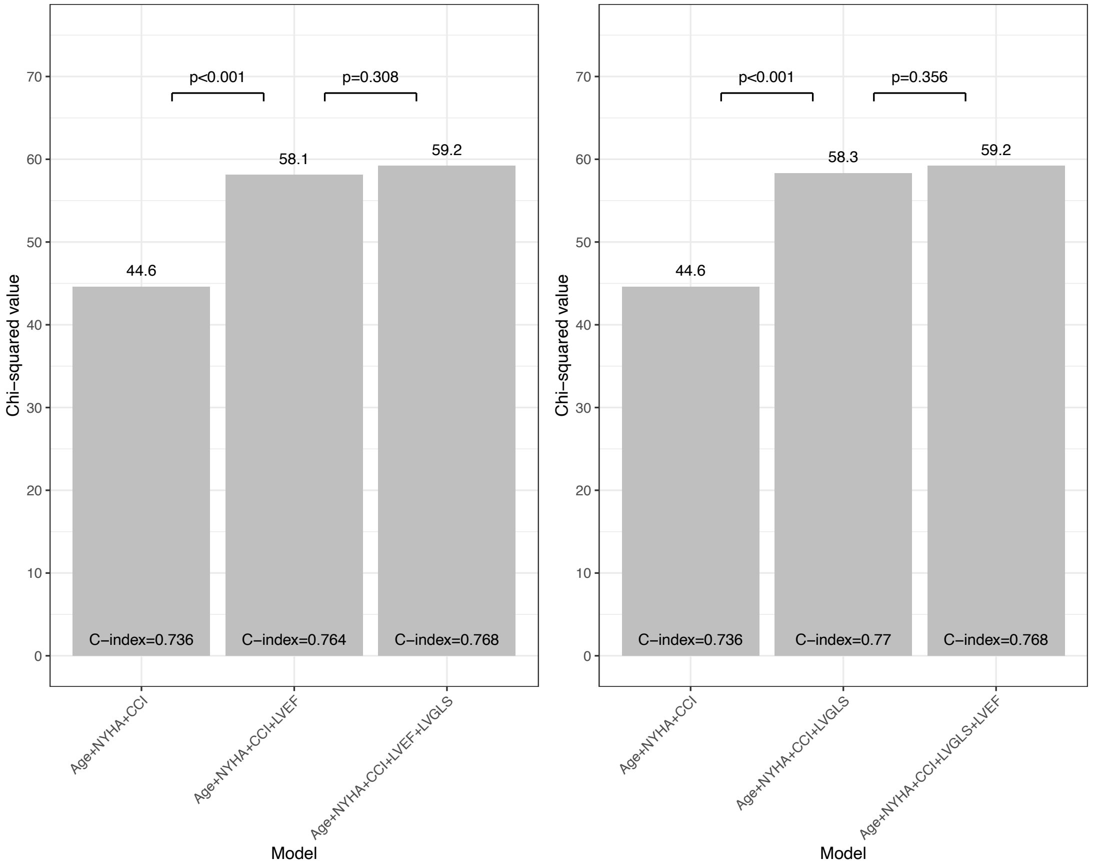

(A) Addition of LVEF (LVGLS) on the initial model that included age, Charlson comorbidity index (CCI), and NYHA classification significantly increased C-index. (B) Further addition of LVGLS (LVEF) did not show an incremental value over initial model + LVEF (LVGLS).

Both LVEF and LVGLS were derived by fully automated analysis.

Supplemental Figure S8. Reclassification tables by addition of LVGLS (LVEF) on the initial model with LVEF (LVGLS).

A: Addition of LVGLS

Reclassification table

Outcome: absent

Updated Model<br>Initial Model	[0,0.0479)	[0.0479,0.114)	[0.114,0.21)	[0.21,1]	% reclassified
[0,0.0479)	95	6	0	0	6
[0.0479,0.114)	6	93	2	0	8
[0.114,0.21)	0	5	73	5	12
[0.21,1]	0	0	7	66	10

Outcome: present

Updated Model<br>Initial Model	[0,0.0479)	[0.0479,0.114)	[0.114,0.21)	[0.21,1]	% reclassified
[0,0.0479)	3	1	0	0	25
[0.0479,0.114)	0	3	1	0	25
[0.114,0.21)	0	2	17	3	23
[0.21,1]	0	0	1	32	3

Combined Data

Updated Model<br>Initial Model	[0,0.0479)	[0.0479,0.114)	[0.114,0.21)	[0.21,1]	% reclassified
[0,0.0479)	98	7	0	0	7
[0.0479,0.114)	6	96	3	0	9
[0.114,0.21)	0	7	90	8	14
[0.21,1]	0	0	8	98	8

NRI(Categorical) [95% CI]: 0.0457 [-0.0471 - 0.1385] ; p-value: 0.33422
NRI(Continuous) [95% CI]: 0.1632 [-0.1038 - 0.4301] ; p-value: 0.23088
IDI [95% CI]: 0.0017 [-0.0038 - 0.0073] ; p-value: 0.54066

B: Additon of LVEF

Reclassification table

Outcome: absent

Updated Model<br>Initial Model	[0,0.0493)	[0.0493,0.109)	[0.109,0.216)	[0.216,1]	% reclassified
[0,0.0493)	94	7	0	0	7
[0.0493,0.109)	10	83	7	0	17
[0.109,0.216)	0	6	75	4	12
[0.216,1]	0	0	10	62	14

Outcome: present

Updated Model<br>Initial Model	[0,0.0493)	[0.0493,0.109)	[0.109,0.216)	[0.216,1]	% reclassified
[0,0.0493)	3	1	0	0	25
[0.0493,0.109)	0	4	1	0	20
[0.109,0.216)	0	0	19	1	5
[0.216,1]	0	0	3	31	9

Combined Data

Updated Model<br>Initial Model	[0,0.0493)	[0.0493,0.109)	[0.109,0.216)	[0.216,1]	% reclassified
[0,0.0493)	97	8	0	0	8
[0.0493,0.109)	10	87	8	0	17
[0.109,0.216)	0	6	94	5	10
[0.216,1]	0	0	13	93	12

NRI(Categorical) [95% CI]: 0.0223 [-0.062 - 0.1067] ; p-value: 0.60374
NRI(Continuous) [95% CI]: 0.0961 [-0.1709 - 0.3632] ; p-value: 0.48051
IDI [95% CI]: 0.0026 [-0.0047 - 0.01] ; p-value: 0.48202

(A) Addition of the left ventricular global longitudinal strain (LVGLS) on the initial model which included age, Charlson comorbidity index, NYHA functional class, and LV ejection fraction (LVEF) did not improve net reclassification improvement (NRI) and integrated discrimination improvement (IDI). (B) Addition of the LVEF on the initial model which included age, Charlson comorbidity index, NYHA functional class, and LVGLS did not improve NRI and IDI.

Supplemental Figure S9. Kaplan-Meier curves according to the combination of cutoff values of LVEF and LVGLS derived by CMR feature-tracking analysis.

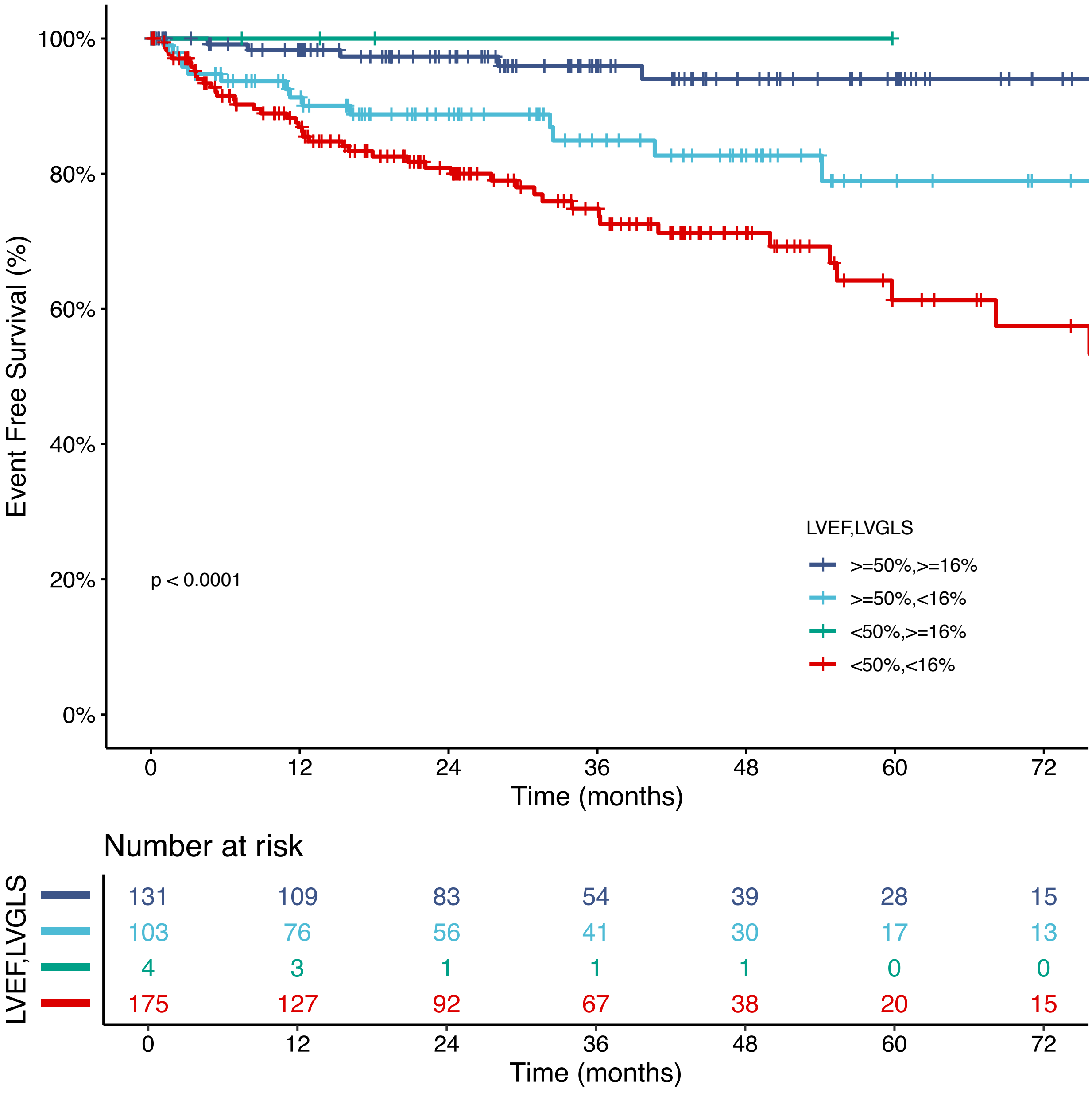

Cutoff values are 50% in LVEF and 16% in LVGLS.

Supplemental Figure S10. Nested Cox proportional hazard models for cardiac events (CMR feature-tracking).

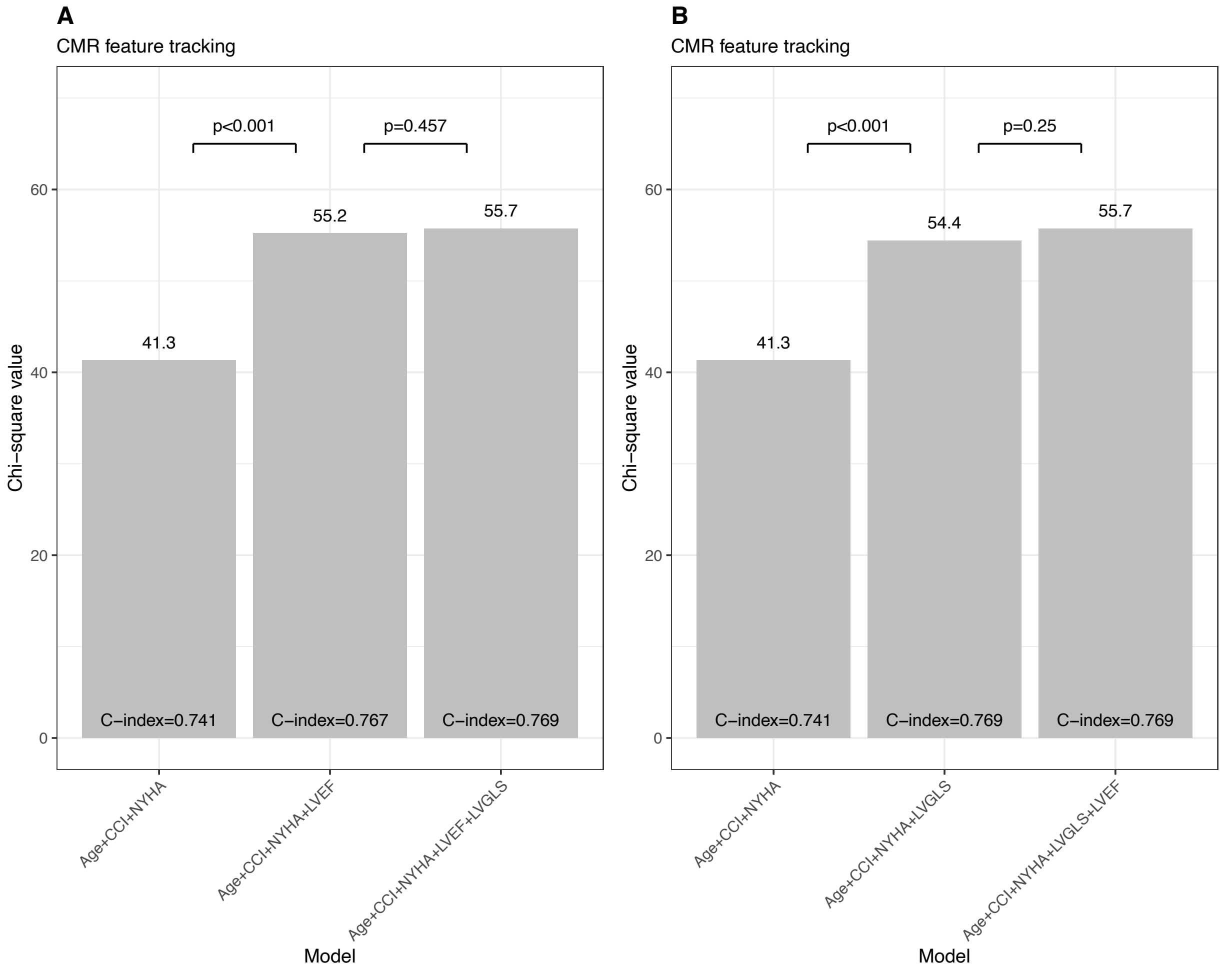

Addition of LVEF (LVGLS) on the initial model that included age, Charlson comorbidity index (CCI), and NYHA classification significantly increased C-index. Further addition of LVGLS (LVEF) did not show an incremental value over initial model + LVEF (LVGLS).

Both LVEF and LVGLS were derived by CMR feature-tracking analysis.

Supplemental Video. Demonstration of fully automated speckle-tracking analysis for left ventricular ejection fraction and global longitudinal strain.

After selecting three apical images, one click for the AutoStrain LV starts to proceed fully automated analysis to provide global longitudinal strain within 10 seconds. On LVEF tab on the right panel, LV volumes and LVEF are also provided.
